# Supplementary material for: Mouse genome-wide association and systems genetics identifies Lhfp as a regulator of bone mass
Source: PLoS Genet. 2019 May 1;15(5):e1008123. doi: 10.1371/journal.pgen.1008123 (PMC6513102; doi:10.1371/journal.pgen.1008123)
Supplement: S1 Table — (DOCX) [file pgen.1008123.s001.docx]

**Supplemental Table 1.** List of primer sequences.

| Oligonucleotide # | Sequence | Comments | Chr 3 Map position (GRCm38.p4 C57BL/6J) |
| --- | --- | --- | --- |
| 1 | CTTC GCC GAC ACG CAG CAA CCC ATC | Oligo for sgDNA cloning | CTTCG + 53043637 - 53043618 bp |
| 2 | AAAC GAT GGG TTG CTG CGT GTC GGC | Oligo for sgDNA cloning | AAAC+53043618 - 53043637 bp +C |
| 3 | GCA TAT GAT ACA CTT GAT GTA CTG GCC | PCR primer for IVT template | NA |
| 4 | GAC TAT CAT ATG CTT ACC GT | Px330 sequencing primer | NA |
| 5 | GAAAT TAA TAC GAC TCA CTA TAG GCC GAC ACG CAG CAA CCC ATC | PCR primer for IVT template | T7 promoter+53043637 - 53043618 bp |
| 6 | CTA CAT ACG CCT TGA AGA GCG | PCR primer for genotyping | 53043194 - 53043214 bp |
| 7 | GGA CTT TGC TGG ATC TCT TAC C | PCR primer for genotyping | 53043849 - 53043828 bp |
| 8 | CCA TTA CAA CCA AGC ATC AGA AGA | Sequencing primer for genotyping | 53043785 - 53043764 bp |
| 9 | TCG GAA CTC ATC TCC AGG AC | qPCR primer for transcripts | 53043634 - 53043653 bp |
| 10 | AGC CAT CCA TGT ACA CAG CA | qPCR primer for transcripts | 53260570 - 53260551 bp |
