# Supplementary material for: Mouse genome-wide association and systems genetics identifies Lhfp as a regulator of bone mass
Source: PLoS Genet. 2019 May 1;15(5):e1008123. doi: 10.1371/journal.pgen.1008123 (PMC6513102; doi:10.1371/journal.pgen.1008123)
Supplement: S1 Fig — (DOCX) [file pgen.1008123.s002.docx]

**Supplemental Figure 1.** Impact of each CRISPR/Cas9 induced mutation on *Lhfp* protein sequence.

Wild Type MASSLTCTGVIWALLSFLSAATSCVGFFMPYWLWGSQLGKPVSFGTFRRCSYPVHDESRQ 60

4bp deletion MASSLTCTGVIWALLSFLSAATSCVGFFMPYWLWGSQLGKPVSFGTFRRCSYPVHDESRQ 60

4&3bp deletion MASSLTCTGVIWALLSFLSAATSCVGFFMPYWLWGSQLGKPVSFGTFRRCSYPVHDESRQ 60

8bp deletion MASSLTCTGVIWALLSFLSAATSCVGFFMPYWLWGSQLGKPVSFGTFRRCSYPVHDESRQ 60

11bp deletion MASSLTCTGVIWALLSFLSAATSCVGFFMPYWLWGSQLGKPVSFGTFRRCSYPVHDESRQ 60

16bp deletion MASSLTCTGVIWALLSFLSAATSCVGFFMPYWLWGSQLGKPVSFGTFRRCSYPVHDESRQ 60

Wild Type MMVMVEECGRYASFQGIPSTEWRICTIVTGLGCGLLLLVALTALMGCCVSELISRTVGRV 120

4bp deletion MMVMVEECGRYASFQGIPSTEWRICTIVTGLGCGLLLLVALTALiaacrnsspglSTOP

4&3bp deletion MMVMVEECGRYASFQGIPSTEWRICTIVTGLGCGLLLLVALTAvaacrnsspglSTOP

8bp deletion MMVMVEECGRYASFQGIPSTEWRICTIVTGLGCGLLLLVALTALMrvgthlqdcrksgwg

11bp deletion MMVMVEECGRYASFQGIPSTEWRICTIVTGLGCGLLLLVALTALrvgthlqdcrksgwgn

16bp deletion MMVMVEECGRYASFQGIPSTEWRICTIVTGLGCGLLLLVALaacrnsspglSTOP

Wild Type AGGIQFLGGLLIGAGCALYPLGWDSEEVRQTCGYISGQFDLGKCEIGWAYYCTGAGAAAA 180

4&3bp deletion

8bp deletion npvpggladwcwlcplplglgqSTOP

11bp deletion pvpggladwcwlcplplglgqSTOP

16bp deletion

Wild Type MLLCTWMACFSGKKQKHYPY 200

Lowercase letters represent the continuation of the translatable open reading frame after the location of the CRISPR/Cas9-induced mutations. These residues are not part of the wild-type *Lhfp* protein.
